# Book Notices

**Published:** 1895-02

**Authors:** 


### Book Notices.

---

A SYSTEM OF LEGAL MEDICINE. By Allan McLane Hamilton, M. D., Consulting Physician to the Insane Asylums of New York City, etc., etc., and Lawrence Godkin, Esq., of the New York Bar, with the collaboration of Prof. James T. Babcock, Lewis Balch, M. D., Judge S. E. Baldwin, Louis E. Binsse, Esq., C. F. Bishop, Esq., A. T. Bristow, M. D., B. F.

Cardozo, Esq., C. G. Chaddock, M. D., A. F. Currier, M. D., C. L. Dana, M. D., Geo. Ryerson Fowler, M. D., W. T. Gibb, M. D., W. S. Haines, M. D., F. A. Harris, M. D., W. B. Hornblower, Esq., Chas. Jewett, M. D., P. C. Knapp, M. D., R. C. McMurtrie, Esq., C. K. Mills, M. D., J. E. Parsons, Esq., C. E. Pellew, E. M., Judge C. E. Pratt, W. A. Purrington, Esq., B. Sachs, M. D., F. R. Sturgis, M. D., Brandreth Symonds, M. D., V. C. Vaughan, M. D. In two large royal octavo volumes of about 700 pages each. Illustrated. Price, in substantial cloth binding, per volume, $5.50; in full sheep, uniform law style, per volume, $6.50. Sold by subscription. Orders taken only for the complete work. E. B. Treat, Publisher, 5 Cooper Union, New York City. Vol. II.

This volume, which completes this most admirable "system," contains 738 pages, with 84 illustrations, and treats of a variety of subjects of the greatest interest to the student of legal medicine. The opening chapter, by Wm B. Hornblower, Esq., of the New York bar, is on "The Duties and Responsibilities of Medical Experts," and no physician, who has been called on to testify before a court, can fail to be interested in this chapter, and every physician should familiarize himself with the rules here laid down for his guidance in giving testimony before courts of law.

The chapter on "Insanity in its Medico-Legal Bearings,,' is by the able editor, Allan McLane Hamilton, M. D. This is a carefully prepared article covering one hundred and forty-nine pages of this volume. The causes and development of insanity, the different forms of insanity, the responsibility of the insane, the responsibility of those in charge of the insane; in fact, insanity in all of its medico-legal bearings receives due consideration in this article.

"Mental Responsibility of the Insane in Civil Cases," is the subject of a short chapter by Calvin E. Pratt, Justice of New York Supreme Court.

"Insanity and Crime," by B. Sachs, M. D.; "On the Relations of Mental Defect and Disease to Criminal Responsibility," by Louis E. Binsse, are the subjects of two most interesting contributions to this volume.

"Aphasia and other Defects of Speech," is the subject of a valuable contribution by Charles K. Mills, M. D.

Charles L. Dana, M. D., devotes more than sixty pages to the discussion of the now popular subject of "The Traumatic Neuroses: Being a Description of the Chronic Nervous Disorders that follow Shock and Injury." This subject, under the names of railway spine, railway brain, traumatic neurasthenia, traumatic hysteria, concussion of the spine, etc., has received much attention during the past few years, and Dr. Dana advances some original ideas on the subject, and makes of it a very interesting and instructive chapter.

The following list of subjects next claim our attention:

"The Effects of Electric Currents of High Power upon the Human Body," by Allan McLane Hamilton, M. D., and George De Forrest Smith, M. D.; "Accident Cases," by Lawrence Godkin, Esq.; "Mental Distress as an Element of Damage in Cases to Recover for Personal Injuries," by John E. Parsons, Esq.; "Feigned Diseases of the Mind and Nervous System," by Phillip Coombs Knapp, M. D.; "Birth, Sex, Pregnancy, and Delivery," by Andrew F. Currier, M. D.; "Abortion and Infanticide," by Charles Jewett, M. D.; "Genito-Urinary and Venereal Affections in their Medico-Legal Relations," by F. R. Sturgis, M. D.; "Marriage and Divorce." by Simeon E. Baldwin, LL. D.; "Sexual Crimes," by Charles Gilbert Chaddock, M. D.; "Surgical Malpractice," by George Ryerson Fowler, M. D. The volume closes with an appendix giving a concise epitome of the laws of the different States and territories of the United States which relate to the general care of the insane.

These two volumes, constituting Hamilton's "System of Legal Medicine," are worthy of the highest commendation. The contributors were selected from the highest ranks of the medical and legal professions, and each and every article in the two volumes bears the stamp of a master. The work as a whole demonstrates the possibilities of close application and persistent toil. It is one of the great books of this century. H.

---

A SYSTEM OF GENITO-URINARY DISEASES, SYPHILOLOGY AND DERMATOLOGY. By various authors. Edited by Prince A. Morrow, A. M., M. D., Clinical Professor of Genito-Urinary Diseases, formerly Lecturer on Dermatology in the University of the City of New York; Surgeon to Charity Hospital, etc. With illustrations. In three volumes. Vol. III, Dermatology. Price per volume, cloth, $6.50. D. Appleton & Co., publishers, New York City.

This volume of 976 pages and with 131 illustrations, completes the "System of Genito-Urinary Diseases, Syphilology and Dermatology." Vol. I, of which quite a lengthy review appeared in this JOURNAL some months since, was devoted to the consideration of Genito-Urinary Diseases. Vol. II treated only Syphilology, and Vol. III is an exhaustive treatise on Dermatology. The majority of the leading dermatologists of this country have made contributions to this volume, and the result is one of the most practical and complete works that we have on the subject of diseases of the skin.

The advances made during the past few years in this particular branch of medical science, the improved methods of investigation and persistent research have demonstrated the existence of a number of pathological conditions heretofore unknown, and this has necessitated the introduction of new terms to convey a correct conception of these conditions. No less than forty different diseases are now recognized as distinct clinica lentities, which a few years ago were unknown, or were identified with other dermatoses. Many diseases, the essential nature of which was formerly but imperfectly understood, have, through recent research and bacteriological study, become more clearly comprehended, and the close relation of certain affections, previously regarded as wholly unrelated, has been demonstrated by this same method of investigation. The necessity, then, for a work complete in all essential details, and thoroughly up to date, is quite apparent.

The editor has done excellent work in selecting his collaborators, in arranging and classifying the new material, and in assigning to each subject the space that its importance demands. The book will no doubt be adopted as one of the principal textbooks on dermatology, and it is sufficiently comprehensive to serve as one of the best books of reference. It is a superior work in every respect, and the editor, contributors and publishers are deserving of the highest praise for the high character of their work.

H.

---

Essentials of Refraction, and the Diseases of the Eye. By Edward Jackson, A. M., M. D., Professor of Diseases of the Eye in the Philadelphia Polyclinic and College for Graduates in Medicine; attending Surgeon to Wills Eye Hospital, etc.

PART II.

Essentials of Diseases of the Nose and Throat. By E. B. Gleason, S. B., M. D., Surgeon in charge of the Nose, Throat and Ear Department of the Northern Dispensary of Philadelphia; formerly Assistant in the Nose and Throat Dispensary of the Hospital of the University of Pennsylvania, and Assistant in the Nose and Throat Department of the Union Dispensary, etc., etc. Second edition, revised; 290 pages, 120 illustrations. Price, cloth, $1.00. W. B. Saunders, publisher, 925 Walnut street, Philadelphia.

This volume constitutes No. 14 of Saunders' popular "Question Compends." The purpose of the author to include in this book an exposition of only settled facts and established principles, leaving to larger works on the same subjects the discussion of those questions which are still in doubt, and those purely theoretical, is a very wise one. The capital operations in ophthalmic surgery are but briefly noticed, and lengthy discriptions of the anatomy and physiology of the parts have been omitted. For these points the student is referred to appropriate works on each.

It is purely a practical work, intended to furnish the student and practitioner with the essentials of diagnosis and treatment, and to those who can extend their studies in this direction, a good foundation for their future progress. The book is far above the average "compend" and is worthy of careful study. H.

---

A MANUAL OF HUMAN PHYSIOLOGY, Prepared with Special Reference to Students of Medicine. By Joseph H. Raymond, A-M., M. D., Professor of Physiology and Hygiene in the Long Island College Hospital, and Director of Physiology in the Hoagland Laboratory. 382 pages, with 102 illustrations and four full-page colored plates. Price, cloth, $1.25. W. B. Saunders, Publisher, 925 Walnut street, Philadelphia. 1894.

The author deals with the main facts of human physiology from the standpoint of a teacher. His twenty year's experience as a teacher of physiology to medical students has led him to the conclusion that in the short time allotted to the study of physiology in medical schools, students can assimilate only the main facts and principles of this important branch of medicine; hence the necessity for a work dealing with only the essentials on this subject. There is no denying the fact, that the larger text-books, from the vast amount of matter contained, and the lengthy discussion on abstruse subjects, are confusing to the average medical student, and especially to those who are attending their first course of lectures. The author of this volume has endeavored to supply a work, short and practical, yet complete in essentials, and peculiarly adapted to the use of the medical student, and in this he has succeeded well. The book is well written throughout and is profusely illustrated. H.

---

THE NURSES' DICTIONARY OF MEDICAL TERMS AND NURSING TREATMENT. Compiled for the use of nurses and containing descriptions of the principal medical and nursing terms and abbreviations, instruments, drugs, diseases, accidents treatment, pysiological names, operations, foods, appliances, etc., etc., encountered in the ward or sick room. By Honnor Morten, author of "Sketches of Hospital Life," "How to Become a Nurse," etc. Second edition, 139 pages. Price, cloth, $1.00. Publishers—W. B. Saunders, 925 Walnut street, Philadelphia; The Scientific Press (limited), 128 Strand, London, W. C. All rights reserved.

This dictionary is intended for the especial use of the nurse at the bed side as a temporary reference, and in this capacity it will serve an excellent purpose. Such words, terms, instruments, accidents, operations, foods, appliances, etc., as are of most interest to nurses, and a knowledge of which will be found useful to them are considered in this little volume, and in the briefest practical way.

The book will be of much service to nurses, and it possesses two especially valuable features, viz.: simplicity and brevity.

H.

---

Essentials of Anatomy, Including the Anatomy of the Viscera; arranged in the form of questions and answers. Prepared especially for students in medicine, by Charles B. Nancrede, M. D., Professor of Surgery and of Clinical Surgery in the University of Michigan, Ann Arbor; late Senior Surgeon to Episcopal Hospital; late Surgeon to Jefferson Medical College Hospital; late Professor of General and Orthopedic Surgery in Philadelphia Polyclinic, etc., etc. Fifth edition; with an appendix on the osteology of the human body; the whole based on the last edition of Gray's Anatomy. 388 pages; 80 fine illustrations. Price, in cloth, $1.00. W. B. Saunders, publisher, 925 Walnut street, Philadelphia. 1894.

In the short space of six years Prof. Nancrede's Essentials of Anatomy has reached its fifth edition and its fifteenth thousand. It is one of the best and most popular short works on anatomy, and is deservedly popular with medical students. The fine osteological plates that have been added in this edition, increase the worth of the book very materially, and the demand for it will no doubt be even greater than in the past.

H.

---

The Treatment of Naso-Pharyngeal Diseases and Their Aural Sequences. A Lecture delivered at the Missouri Medical College, by H. N. Spencer, A. M., M. D., Professor of Diseases of the Ear. J. B. Lippincott Co., Publishers, Philadelphia, Pa.

This small volume contains a general summary of Prof. Spencer's usual teaching on the subject of naso-pharyngeal diseases before the classes of the Missouri Medical College. He gives his methods of examining the nose, throat and ear, the instruments and appliances necessary to make these examinations, and the proper line of treatment to be instituted for the relief of the various disorders affecting these cavities. The book is thoroughly practical and will be found useful to all, but especially so to the general practitioner. It is printed on nice heavy paper, with flexible covers, and contains a number of illustrations. H.

---

AN ILLUSTRATED MONOGRAPH ON KOLA. Published under the direction of F. E. Stewart, M. D., Ph. G., Director Scientific Department, F. Stearns & Co. Formerly Demonstrator and Lecturer on Materia Medica and Pharmacy, Jefferson Medical College, etc. F. Stearns & Co., Publishers, Detroit, Mich.

This very interesting monograph is a scientific treatise, carefully compiled by scientific and professional men, and is not, as one would suppose, an advertising circular. It is bristling with facts concerning Kola, and one who studies it carefully will come out well posted on this important medicinal agent, its name and synonyms, habitat, history, botany, cultivation, collection and transportation, substitutions and adulterations, chemistry, its active constituents, physiological and therapeutical action, in fact, all about Kola, and what it is good for.

---

THE F. A. DAVIS CO., of Philadelphia, announce that they will issue, early in February, a companion book to Dr. R. von Krafft-Ebing's famous treatise, "Psychopathia Sexualis," entitled "Suggestive Therapeutics in Psychopathia Sexualis," it being a translation of the original by Dr. A. Schrenck-Notzing, of Munich, collaborator with Krafft-Ebing. This book will contain about 325 pages, and be sold by subscription only, at $2.50 per volume, in cloth. It will be of the greatest importance as an authoritative work on suggestion as a therapeutic agent in the hands of the intelligent practitioner.

